# Tunable First-Order Resistorless All-Pass Filter with Low Output Impedance

**DOI:** 10.1155/2014/219453

**Published:** 2014-01-22

**Authors:** Parveen Beg

**Affiliations:** Department of Electronics Engineering, A.M.U., Aligarh 202002, India

## Abstract

This paper presents a voltage mode cascadable single active element tunable first-order all-pass filter with a single passive component. The active element used to realise the filter is a new building block termed as differential difference dual-X current conveyor with a buffered output (DD-DXCCII). The filter is thus realized with the help of a DD-DXCCII, a capacitor, and a MOS transistor. By exploiting the low output impedance, a higher order filter is also realized. Nonideal and parasitic study is also carried out on the realised filters. The proposed DD-DXCCII filters are simulated using TSMC the 0.25 *µ*m technology.

## 1. Introduction

Analog first-order filter design using a variety of active building blocks has been the focus of research for the past several decades. Operational amplifier was the active element of choice during the earlier stages of development. Later, the advent of second generation current conveyors (CCII), differential voltage current conveyor (DVCC), current controlled current conveyor (CCCII), and dual-X current conveyor (DXCCII) signalled the era of voltage-mode and current-mode signal processing. Since then, there has been a significant amount of technical literature available on the subject. Obviously, it is not possible to attempt a thorough review of all the related works. However, a survey of some of the recently published first-order voltage mode all-pass filters (APF) is presented in this section. A variety of circuits are available based on the above-mentioned conveyors [1–22]. The filter in [1–8] is composed of more than one active element while the method proposed in [9–22] employs only a single active element and passive components. The filters in [16, 17] employ four passive components; the filter in [19] employs two to three passive components while the filters designed in [10–13, 15, 20, 21] employ three passive components. Out of these, the filters presented in [11, 20, 21] do not exhibit a low output impedance and hence fall in the category of noncascadable filters while those in [10, 12, 13, 15] have a low output impedance and are classified as cascadable filters. The filters in [1, 3, 4, 18] are resistorless circuits based on one or more active elements. A distinct advantage of the filter presented in [18] is that it employs a single active and a passive element with low output impedance.

However, none of the above-mentioned filters support tuning as a feature except the filter in [1].

The DXCCII presented in [15], even though contains a buffered output, requires an additional current source to obtain a low output impedence terminal, while, in this work, no additional current source is required. The DD-DXCCII is utilized to design a tunable voltage mode cascadable first-order filter.

The single active element all-pass filter presented in this paper exhibits the following features:

- single active element DD-DXCCII,
- single passive element,
- resistorless,
- low output impedance,
- tunable,
- no matching condition.

A detailed comparison of some of the reported single active element voltage mode filters with the proposed filter is shown in Table 1. From this table, it is clear that none of the reported filters exhibit all the above-mentioned features.

The rest of the paper is organized as follows.  Section 2 presents a brief overview of the DD-DXCCII, with buffered output followed by the design and analysis of a new voltage-mode, first-order, all-pass filter section based on a single DD-DXCCII. In Section 3, nonideal and parasitic analysis of the proposed circuit is performed. The feature of easy cascadibility is highlighted in Section 4 while Section 5 presents the results of computer simulations of the proposed circuits using the PSPICE program. Some conclusive remarks appear in Section 6.

## 2. Proposed First-Order All-Pass Filter

The differential difference dual-X current conveyor with buffered output has been proposed as an eight-terminal device characterized by the following port relations:

(1) $$\begin{array}{c} \left[\begin{array}{c} I_{y1}\\ I_{y2}\\ I_{y3}\\ V_{xp}\\ \begin{array}{c} V_{xn}\\ I_{zpi} \end{array}\\ \begin{array}{c} I_{zni}\\ V_{wn} \end{array} \end{array}\right]=\left[\begin{array}{cccccc} 0 & 0 & 0 & 0 & 0 & 0\\ 0 & 0 & 0 & 0 & 0 & 0\\ 0 & 0 & 0 & 0 & 0 & 0\\ 1 & -1 & 1 & 0 & 0 & 0\\ -1 & +1 & -1 & 0 & 0 & 0\\ 0 & 0 & 0 & 1 & 0 & 0\\ 0 & 0 & 0 & 0 & 1 & 0\\ 0 & 0 & 0 & 0 & 0 & 1 \end{array}\right]\left[\begin{array}{c} V_{y1}\\ V_{y2}\\ V_{y3}\\ I_{xp}\\ \begin{array}{c} I_{xn} \end{array}\\ \begin{array}{c} V_{zn} \end{array} \end{array}\right]. \end{array}$$

**Figure 1:**
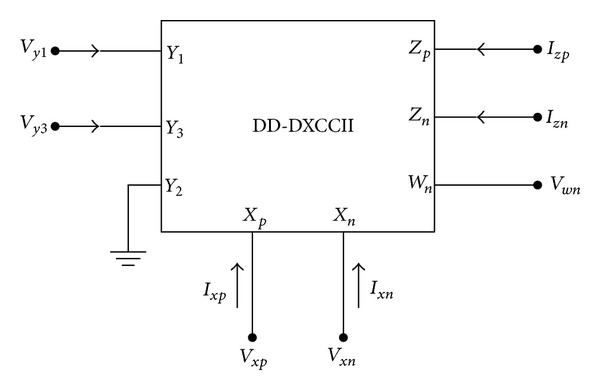
Schematic symbol of DD-DXCCII.

**Figure 2:**
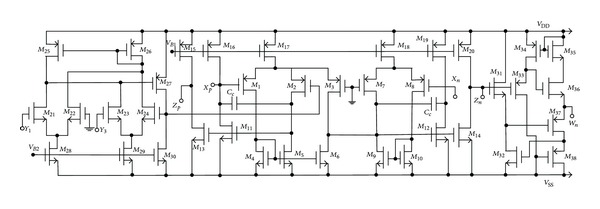
CMOS implementation of DD-DXCCII.

Figure 1 shows the schematic symbol of a DD-DXCCII with buffered output. The CMOS implementation of DD-DXCCII with buffered output at *Z*_*n*_ is shown in Figure 2.

The input side has three terminals (called *Y*_1_, *Y*_2_, and *Y*_3_) and there are two *X*-terminals (*X*_*p*_ and *X*_*n*_). At the output side, there are three terminals *Z*_*p*_, *Z*_*n*_, and *W*_*n*_. For the *Z*_*p*_ terminal, the direction and magnitude of the conveyed current are the same as those of the current flowing in the *X*_*p*_-terminal whereas for the *Z*_*n*_ terminal, the current is the same as in the *X*_*n*_-terminal. The voltage that appears at terminal *W*_*n*_ is equal to the voltage at *Z*_*n*_.

**Figure 3:**
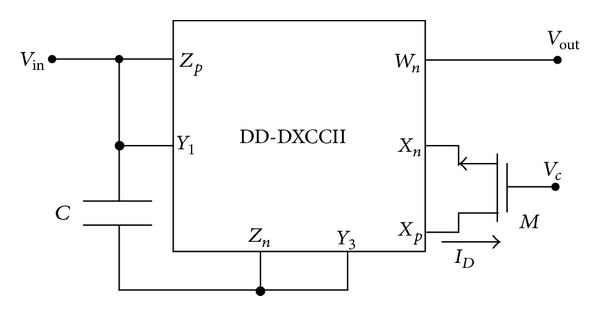
Proposed tunable first-order all-pass filter.

The proposed tunable voltage-mode first-order all-pass filter section is shown in Figure 3. As can be seen, the circuit has minimal complexity and employs a single DD-DXCCII, a single capacitor, and an NMOS transistor biased in the triode region.

The transfer function of an all-pass filter can be given as

(2) $$\begin{array}{c} T_{AP}=\frac{V_{\text{out}}}{V_{\text{in}}}=\frac{s-2/R_{M}C}{s+2/R_{M}C}, \end{array}$$

where *R*_*M*_ is the resistance of the MOSFET transistor (*M*) in Figure 3 and is given by

(3) $$\begin{array}{c} R_{M}={\left[\mu_{n}C_{ox}\left(\frac{W}{L}\right)\left(V_{C}-V_{T}\right)\right]}^{-1}, \end{array}$$

where *μ*_*n*_, *C*_*ox*_, *V*_*T*_, *W*, and *L* are the surface mobility, oxide capacitance, threshold voltage, channel width, and length of MOS. From (2) the pole frequency *ω*_0_ can be expressed as

(4) $$\begin{array}{c} \omega_{0}=\frac{2}{R_{M}C}. \end{array}$$

From (4) it is clear that the pole frequency of the all-pass filter can be tuned by varying the resistance (*R*_*M*_) of the triode MOS resistor (*M*). The phase angle of the all-pass filter can be expressed as

(5) $$\begin{array}{c} ∠\phi =\pi -2\tan^{-1}\left(\frac{\omega CR_{M}}{2}\right). \end{array}$$

## 3. Nonideal Study

### 3.1. Effect of Nonideal Transfer Gain

The port relations defining the DD-DXCCII, as given in (1), correspond to an ideal device in which the current and voltage conveying processes are deemed perfect. For a more realistic understanding of the operation of the circuit of Figure 3, the nonidealities associated with the DD-DXCCII need to be taken into consideration. The nonideal port relationship can be expressed as

(6) Vxp=βp1Vy1−βp2Vy2+βp3Vy3,Vxn=βn1Vy1−βn2Vy2+βn3Vy3,Izp=αpIxp,  Izn=αnIxn,Vwn=γnVzn,

where *β*_*pi*_ (*β*_*ni*_) is the voltage transfer gain from the *Y*_*i*_ port to *X*_*p*_ (*X*_*n*_) port, where *i* = 1 to 3, *α*_*p*_ (*α*_*n*_) is the current transfer gain from the *X*_*p*_ port to *Z*_*p*_ port (*X*_*n*_ port to *Z*_*n*_ port) and *γ*_*n*_ is the voltage transfer gain from *Z*_*n*_ port to *W*_*n*_ port (ideally, these transfer gains are unity in magnitude).

Using (6) the ideal transfer functions of the AP filter section, given in (2), yield the following nonideal transfer function:

(7) $$\begin{array}{c} T_{\text{AP},\text{nonideal}}=\frac{V_{\text{out}}}{V_{\text{in}}}=\frac{s-\left(2\alpha_{p}\beta_{1}/R_{M}C\right)}{s+\left(2\alpha_{p}\beta_{3}/R_{M}C\right)}. \end{array}$$

From (7) the nonideal pole frequency can be expressed as

(8) $$\begin{array}{c} \omega_{0,\text{nonideal}}=\frac{2\alpha_{p}\beta_{3}}{R_{M}C}. \end{array}$$

Equation (8) shows that, due to nonideal voltage and current transfer gain, the pole frequency does get affected. The sensitivity analysis shows that the pole frequency due to the nonidealities is unity in magnitude.

### 3.2. Effect of Parasitics

The parasitics associated with the actual DD-DXCCII are the same as those of the DXCCII [23]. In Figure 3, *Y*_1_ and *Z*_*p*_ port parasitics are in parallel; that is, *R*_*y*1_//*R*_*zp*_//*C*_*y*1_//*C*_*zp*_, *Y*_3_, and *Z*_*n*_ port resistances and capacitances are also in parallel, that is, *R*_*y*3_//*R*_*zn*_//*C*_*y*3_//*C*_*zn*_. Also, the *X*_*p*_ and *X*_*n*_ parasitics, that is, *R*_*xp*_ and *R*_*xn*_, merge with the resistance of triode MOSFET. The proposed circuit is reanalyzed by taking into account the above parasitics (assuming *R*_*M*_ ≫ (*R*_*xp*_ + *R*_*xn*_)). The nonideal transfer gain due to parasitics then becomes

(9) $$\begin{array}{c} T_{\text{AP},\text{parasitic}}=\frac{V_{\text{out}}}{V_{\text{in}}}=\left(\frac{C+C^{\prime }}{C}\right)\left(\frac{s-2/R_{M}\left(C+C^{\prime }\right)}{s+2/R_{M}C}\right), \end{array}$$

where *C*′ = *C*_*y*1_//*C*_*zp*_ and since *C* ≫ *C*′, the transfer function reduces to

(10) $$\begin{array}{c} T_{\text{AP},\text{parasitic}}=\frac{V_{\text{out}}}{V_{\text{in}}}=\left(\frac{s-2/R_{M}C}{s+2/R_{M}C}\right). \end{array}$$

From (10) it is clear that the pole frequency is unaffected in the presence of parasitics.

## 4. Higher Order All-Pass Filter

**Figure 4:**
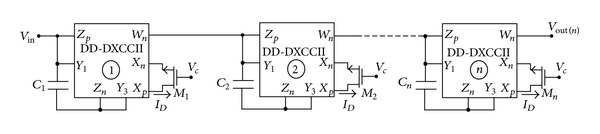
Proposed tunable *n*th order all-pass filter.

It is well known that higher order filters exhibit a larger rate of phase change at constant magnitude when compared to a first-order filter. They can also be used as a group delay equalizer in video and communication applications. These features are the motivating factor behind realizing a higher order filter in this work. The proposed *n*th order APF filter is presented in Figure 4 which is obtained by cascading *n*-stages of the first-order filter described in Figure 3. The proposed filter employs *n*-stages of DD-DXCCII and *n*-capacitors and MOS resistors operating in the triode region.

Analysis of the above circuit yields the following transfer function:

(11) TAP,nth  order=VoutVin=(s−2/RM1C1s+2/RM1C1)×(s−2/RM2C2s+2/RM2C2)⋯(s−2/RMnCns+2/RMnCn).

From (11) the pole frequency can be expressed as

(12) ω0,nth  order =(2n(RM1·RM2⋯RMn)·(CM1·CM2⋯CMn))1/n.

## 5. Design and Verification

**Figure 5:**
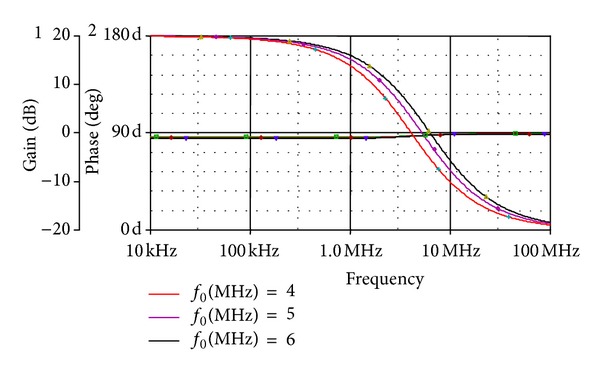
Frequency response showing gain and phase of APS of Figure 3.

**Figure 6:**
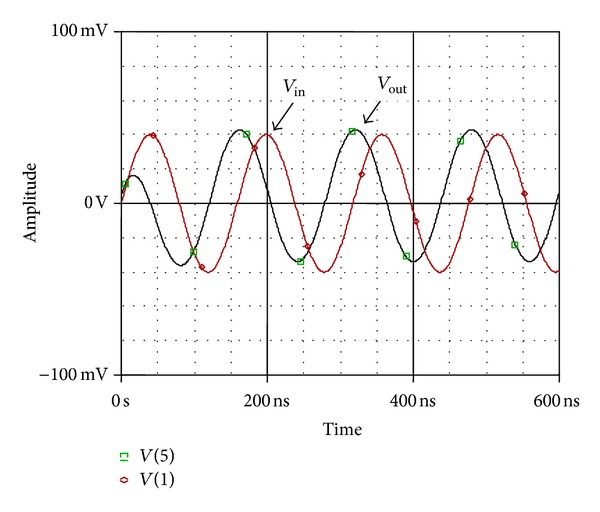
Time domain response of APS at 6 MHz.

**Figure 7:**
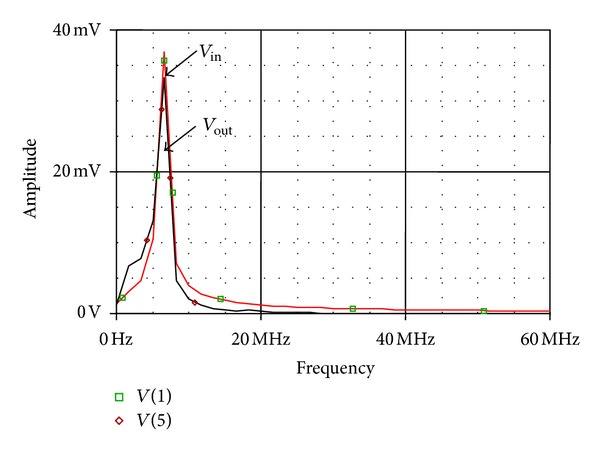
Fourier spectrum of input and output at 6 MHz.

The performance of the first-order all-pass shown in Figure 3 was verified using PSPICE program. Supply voltages were kept at ±1.25 V. The proposed filter was designed with *C* = 10  pF and gate control voltages are *V*_*C*_ = 0.8 V, 1.0 V, and 1.2 V. The gain and phase plot is shown in Figure 5 which shows the variation in pole frequency at different control words. As can be seen, the pole frequency is varied from 4 MHz, 5 MHz, and 6 MHz at *V*_*C*_ = 0.8 V, 1.0 V, and 1.2 V, respectively. The time domain response of the proposed all-pass filter as shown in Figure 6 is obtained by applying a sine wave of 80 mV peak-to-peak amplitude at 6 MHz. The output is 88.9° phases shifted, which corresponds well with the theoretical value of 90°. The total harmonics distortion was found to be 1% at pole frequency of 6 MHz. The Fourier spectrum of input and output waveform is also shown in Figure 7.

**Figure 8:**
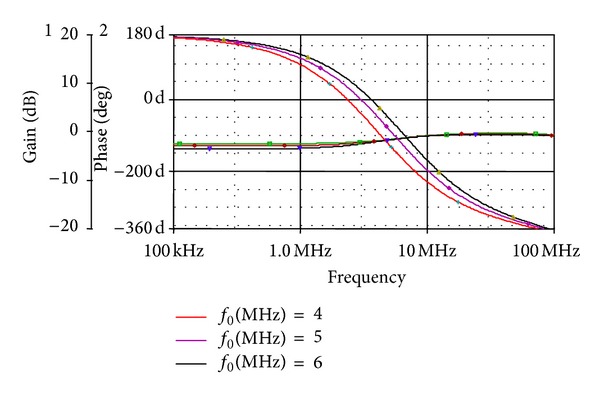
Frequency response showing gain and phase of 3rd order APS of Figure 4.

**Figure 9:**
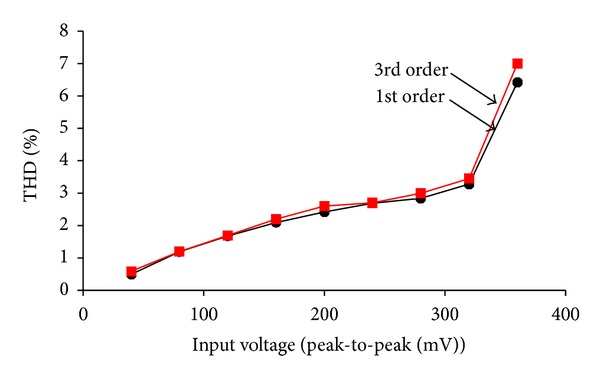
THD variation of 1st and 3rd order filter.

The circuit proposed in Figure 4 is verified for a third-order filter; that is, *n* = 3. The filter was designed at *C* = 10 pF at different gate control voltages of the MOS transistor; that is, *V*_*C*_ = 0.8 V, 1.0 V, and 1.2 V. Simulated gain and phase response at different pole frequencies is shown in Figure 8. The pole frequencies are found to be 4 MHz, 5 MHz, and 6 MHz at a phase of −90°. The variation in pole frequency is obtained by varying the control word. The total harmonics distortion (THD) variation of first- and third-order filter is shown in Figure 9, which shows that the THD variation is around 3% up to 300 mV of the variation in the input signal amplitude.

## 6. Conclusion

In this paper a new active element, that is, DD-DXCCII with buffered output, is presented. By employing DD-DXCCII a new voltage mode tunable resistorless all-pass filter using single passive element is realized. The proposed filter does not require any matching condition. The filter has low output impedance which is elaborated by designing a third-order filter. Nonidealities of the active element along with parasitics are also considered, so as to evaluate the proposed filter. The filter reported in [23, 24] is a current mode all-pass filter while the filter presented in this work is a voltage mode all-pass filter.

## Figures and Tables

**Table 1:** Comparison of single active element based proposed and existing first-order circuits.

Ref no.	Active element	No. of passive components	Resistorless	High input impedance	Low output impedance	Supply voltages	Matching condition required	Tunable	Highest operating frequency
[9]	CCII	5	No	Yes	No	—	Yes	No	~10 KHz
[10]	FDCCII	3	No	Yes	Yes	±3.3 V	Yes	No	1.59 MHz
[11]	CCII	3	No	Yes	No	—	Yes	No	—
[12]	UVC	3	No	Yes	Yes	±2.5 V	Yes	Yes	1.17 MHz
[13]	CBTA/ CCCDBA	3	No	No	Yes	±2.5 V	No	No	119 KHz
[14]	DDCC	2	No	No	No	±2.5 V	No	No	1.59 MHz
[15]	DXCCII	2/3	No	Yes	Yes	±2.5 V	No	No	25 MHz
[16]	CCIII	4	No	No	No		Yes	No	1 KHz
[17]	DXCCII	4	No	Yes	No	±1.25 V	Yes	No	1.59 MHz
[18]	VD-DIBA	1	Yes	Yes	Yes	—	No	No	318 KHz
[19]	ICCII	2	No	No	No	±2.5 V	Yes	No	370 KHz
[20]	CCII	3	No	Yes	No	±2.5 V	Yes	No	1 MHz
[21]	CCII	3	No	No	No	—	Yes	No	159 KHz
[22]	FDCCII	2	No	Yes	Yes	±3 V	No	No	3.11 MHz

Proposed	DDDXCCII	1	Yes	No	Yes	±1.25 V	No	Yes	4–6 MHz
